# Disease Surveillance Investments and Administration: Limits to Information Value in Pakistan Polio Eradication

**DOI:** 10.1111/risa.13580

**Published:** 2020-08-21

**Authors:** Ryan P. Scott, Alison C. Cullen, Guillaume Chabot‐Couture

**Affiliations:** ^1^ Daniel J. Evans School of Public Policy and Governance University of Washington Seattle WA USA; ^2^ Political Science Colorado State University Fort Collins CO USA; ^3^ Institute for Disease Modeling P.O. Box 23350 Seattle WA 98102

**Keywords:** Disease surveillance, polio, value of information

## Abstract

In Pakistan, annual poliovirus investment decisions drive quantities of supplemental immunization campaigns districts receive. In this article, we assess whether increased spending on poliovirus surveillance is associated with greater likelihood of correctly identifying districts at high risk of polio with assignment of an elevated “risk ranking.” We reviewed programmatic documents from Pakistan for the period from 2012–2017, recording whether districts had been classified as “high risk” or “low risk” in each year. Through document review, we developed a decision tree to describe the ranking decisions. Then, integrating data from the World Health Organization and Global Polio Eradication Initiative, we constructed a Bayesian decision network reflecting investments in polio surveillance and immunization campaigns, surveillance metrics, disease incidence, immunization rates, and occurrence of polio cases. We test these factors for statistical association with the outcome of interest—a change in risk rank between the beginning and the end of the one‐year time period. We simulate different spending scenarios and predict their impact on district risk ranking in future time periods. We find that per district spending increases are associated with increased identification of cases of acute flaccid paralysis (AFP). However, the low specificity of AFP investment and the largely invariant ranking of district risk means that even large increases in surveillance spending are unlikely to promote major changes in risk rankings at the current stage of the Pakistan polio eradication campaign.

## 1 INTRODUCTION

Poliomyelitis remains an endemic disease in Pakistan, Nigeria, and Afghanistan, with the final stages of disease eradication proving difficult. In Pakistan, as disease incidence trended downward from 2012 to 2017, identifying the probability of incidence in diverse parts of the country became increasingly challenging (Roberts, 2018). Subsequently, increases of incidence throughout Pakistan resulting from programmatic and social challenges have further challenged eradication efforts (Ashgar, 2020). Evaluating failures and successes for the declining incidence period thus remains critical to planning for future polio eradication efforts.

While vaccination for polio has proven effective globally, eradication of polio is challenged by the rare presentation of clinical symptoms among infections. Approximately 1 in 200 polio infections create acute flaccid paralysis (AFP), a clear and observable effect (Nathanson & Kew, 2010). Traditional poliovirus eradication efforts involve conducting vaccination programs that aim to achieve greater than 80% vaccination coverage of children under 5 years of age in endemic countries by conducting supplemental immunization campaigns (SIA) and immunization mop‐ups in outbreak areas (Nathanson & Kew, 2010). While this has been globally effective, access (Closser, 2010; Verma, Jimenez, Tangermann, Subramanian, & Razak, 2018), politics (Nishtar, 2010), local practices (Khan et al., 2015), reduced vaccine coverage and efficacy (Patriarca, Wright, & John, 1991), effectiveness (Grassly et al., 2006), violence, and false rumors have resulted in persistent challenges to complete eradication (Reardon, 2011; Riaz & Rehman, 2013).

In this article we evaluate a specific challenge in the polio endgame. Specifically, what are the tradeoffs between allocating program funding towards disease surveillance compared to immunization? We aim to test how improved knowledge about where and how to immunize might provide more effective spending of immunization resources. To answer this question, we utilize historical budget data from the 2012–2017 to evaluate how budget allocations to disease relate to changes in administrative decision making about polio eradication efforts.

## 2 RATIONALE

### 2.1 Polio Surveillance in A Global Landscape

Polio surveillance is a multifaceted endeavor. Previous studies have assessed two major components—the value of polio laboratory networks (De Gourville, Duintjer Tebbens, Sangrujee, Pallansch, & Thompson, 2006; Mulders et al., 2017), and environmental surveillance via sewage sampling (Hovi et al., 2012; Johnson Muluh et al., 2016). While resources for eliminating polio are considerable—in 2016 the Global Polio Eradication Initiative (GPEI) spent $1.1 billion USD, not including country‐level public health spending, most of these funds go towards running immunization campaigns, procuring immunizations, and providing technical assistance (GPEI‐Annual Expenditure Reports, n.d.).

Global expenditures on surveillance in 2016 were $67 million, or just above 6% of the $1.1 billion USD total, despite the fact that most recent studies of poliovirus in Pakistan have identified improved methodologies of surveillance as being critical to eradication efforts (Roberts, 2018; Kroiss et al., 2018; GPEI n.d.). Nevertheless, spending on surveillance in Pakistan mirrors the global pattern of focus on immunization campaigns. In 2014, Pakistani district budgeting for surveillance was just 3.5% of the amount budgeted for supplemental immunization campaigns. Previous estimates indicated that lab costs, excluding fieldwork, for AFP surveillance programs cost upwards of $43 million across the globe (2016 estimate) (Duintjer Tebbens, Diop, Pallansch, Oberste, & Thompson, 2019). Investments in surveillance might improve knowledge of true incidence, but these efforts do not directly impact incidence as immunization does. Yet, such efforts are much more costly: in 2014 immunization spending in Pakistani districts alone totaled over $156 million.

Repeated vaccination efforts can lead to fatigue and resistance within a population, thus reducing the effectiveness of vaccination campaigns. In this context, focusing vaccination proactively could make them more effective and could thus reduce the burden of repeated vaccination campaigns; however, reliance on past cases means such actions are predominantly reactive (Closser, 2010; Khan & Sahibzada, 2016). This is not to say reactive surveillance is not potentially of value, but instead that it is limited in its ability to prevent new outbreaks. Surveillance cannot replace immunization—immunization in polio negative areas is certainty still necessary—but it does provide second‐order decision value about where to target interventions and can potentially improve understanding of the scale of a problem, facilitating proactive campaigns. Like any source of information in decision making, surveillance does nothing to change the underlying characteristics but instead informs how decisionmakers parameterize those decisions.

One of the challenges in allocating immunization program resources is knowing where to target supplemental campaigns and mop‐up campaigns. With fewer than 1 in 100 polio infections presenting clinical symptoms, polio prevalence is uncertain (Nathanson & Kew, 2010). Further, identification of a case after multiple years of no cases has in some instances revealed the extent to which the true prevalence can be unknown without robust surveillance, maintenance, and monitoring (Roberts, 2016). Repeated detections in previously polio‐free regions is considered evidence of reestablished transmission (Duintjer Tebbens & Thompson, 2018; Hadi & Sohail, 2015).

Increasing disease surveillance has the potential to clarify underlying poliovirus circulation patterns and thus potentially disambiguate disease reimportation from a low‐level of endemic circulation. For a program with limited resources for proactive immunization but a need to respond reactively with limited knowledge via supplemental campaigns, this difference can be important (Duintjer Tebbens & Thompson, 2018; Duintjer Tebbens, Pallansch, Wassilak, Cochi, & Thompson, 2016). Other scholarship has studied the impact of political destabilization and Taliban resistance on the ability of polio disease workers to vaccinate and conduct surveillance (Guarino, Voorman, Gasteen, Stewart, & Wenger, 2017; Molodecky et al., 2017; Verma et al., 2018). These papers provide useful informative background about the requirements for polio immunization resources and the potential threats created by instability in the region. Additional scholarship to connect polio funding decisions, such as where supplemental immunization campaigns should be targeted, to the organization of emergency operation center's (EOC) polio eradication initiative (PEI) and the GPEI would be complementary.

To prioritize resource allocations for immunization campaigns and surveillance, PEI and GPEI utilize a risk ranking strategy whereby districts are classified as endemic, high, and low risk, with the majority of resources targeted at endemic and high‐risk districts. This polio outbreak risk ranking is a combination of expert knowledge and quantitative risk modeling (Mercer et al., 2017; Molodecky et al., 2017; Upfill‐Brown, Lyons, Hu, Eckhoff, & Chabot‐Couture, 2014). Typically, a fixed fraction of all subnational areas, for example, districts, will receive additional vaccination campaigns and more technical assistance based on this risk ranking.

### 2.2 Administrative Decision Processes and Implications

Program administrators decide where resources will be allocated given best available information about current disease incidence and forecasted incidence. Decision making in regards to where to target resources are based on a qualitative risk assessment framework developed by WHO (2014) that divides countries into high, medium and low risk categories based on numeric synthesis of indicators describing surveillance quality, disease susceptibility, and surveillance products (Lowther et al., 2013). Alternatively, risk of disease outbreak can be modeled. This can potentially allow targeting of resources to areas with highest risk of outbreak in a more precise manner based on synthesis of multiple data points at a subnational level (Mercer et al., 2017). This goes beyond previous risk assessment products of WHO because it can facilitate decision making at a subnational level. Modeling of subnational polio risk has recently been used to target spending in Pakistan during both the periods of declining incidence and reemergence post 2017 (Mercer et al., 2017).

Developed in 2014, Pakistan used data at the district level to identify reservoirs, high‐risk areas and low‐risk areas for targeting interventions.^1^ SIA were allocated based on a two part modeling strategy that predicted probability of cases and likelihood of importation^2^. Because the risk was derived from disease models (Mercer et al., 2017), SIA allocation were directly determined by available information including:

- population immunity,
- routine immunization zero‐dose fraction,
- under‐immunized fraction,
- recent Wild Poliovirus Type‐1 (WPV‐1) cases,
- recent neighboring WPV‐1 cases,
- recent neighboring compatible cases, and
- remaining risk based on total historical WPV cases. (NEAP, 2015, p. 29)

Risk tiers per district based on this model are then included in the national emergency action plan which is produced annually. Accordingly, the risk rankings reflect planned interventions not necessarily the on‐the‐ground realities of where immunizations occur as unexpected outbreaks may result in additional campaigns—these unexpected changes are instead reflected in changes in updates to the rank during the following time period.

The difficulties in observing polio and social barriers to surveillance creates a challenge. Incorrect or incomplete information could lead to an incorrect risk ranking of districts and thus limit programmatic effectiveness, lengthening the time to eradication, and increasing the total cost. Numerous districts change between risk categorization levels, flipping from low priority to high priority after a new case is discovered—this is indicative of a reactive response and an incorrect original ranking. We describe this further in section 3.2. In practice, flipping from low to high priority means districts that did not have supplemental immunization activity for years were reentered into supplemental surveillance activities. Continuation of the supplemental activity might have better reduced incidence had the true state of the disease been known. In other cases, risk rankings had to be increased because of social unrest or vaccination fatigue resulting in decreased programmatic efficacy (Verma et al., 2018).

Poliovirus surveillance by way of finding and processing AFP cases is imperfect. First, it can only detect 1 out of 200 poliovirus infections even if it is functioning perfectly. AFP detection is itself a signal of a well‐functioning polio surveillance network. The World Health Organization considers regions with a non‐Polio AFP rate of two or more cases per year per 100,000 children under age 15 years to be adequate for capturing transmission. Second, detecting polio requires the collection and laboratory analysis of two stool samples from each AFP case, within 14 days of paralysis onset. If these stool samples are collected too late, or if they are exposed to excessive heat during transport, it may not be possible to isolate poliovirus and thus these polio cases might be wrongly categorized as non‐Polio. The process of testing AFP case‐derived samples for polio falls under the domain of the global polio laboratory network, a global venue for establishing both whether a case of AFP is positive for polio, and for distinguishing the strain of the virus (De Gourville et al., 2006; Hull & Dowdle, 1997). As a supplement to AFP surveillance, environmental sampling (ES) has demonstrated promise in terms of increased sensitivity of identifying circulating virus in the absence of paralysis cases (Hovi et al., 2012; Huang et al., 2005; Kroiss et al., 2018).

Given that only a proportion of AFP cases are polio cases, and due to the limited geographical coverage of ES programs, funds spent on increasing the number of AFP cases detected or environmental samples processed will not necessarily lead to a different risk ranking or to different prioritization of resources (Keeney & Raiffa, 1993; Nosyk, Sharif, Sun, Cooper, & Anis, 2011). Environmental surveillance provides one potential supplement to AFP use (Manor et al., 1999), but has had relatively few uses within a the SIA planning process in Pakistan (Kroiss et al., 2018; Duintjer Tebbens et al., 2017). Additional spending on vaccination instead of surveillance may not necessarily lead to reductions in polio incidence or the prevention of future polio outbreaks unless these vaccination efforts are focused on areas of greatest need or to known transmission zones. Accordingly, data from past polio surveillance budgets, decision processes, and resulting incidence of disease can inform an assessment of the value of increased spending on surveillance versus immunization.

To date, most modeling of decisions in polio eradication focuses on models of disease and the potential improvements to prediction rather than modeling the administrative processes of decision making and the impact of information within those processes (Duintjer Tebbens & Thompson, 2018; Ghafoor & Sheikh, 2016; Upfill‐Brown et al., 2014; Upfill‐Brown, Voorman, Chabot‐Couture, Shuaib, & Lyons, 2016). A pitfall of modeling the disease without including the decision making system as an active component is that its ability to influence decision may be limited if the on‐the‐ground realities of implementation and the institutional demands of budgeting and planning are not considered; administrative practices can be resistant to change (Ahmad, 2017; Closser, 2010; Ostrom, 1990; Thompson, Tebbens, Pallansch, Wassilak, & Cochi, 2015). Furthermore, administrative budgeting decisions are based on best estimates and expectations, usually defined by incremental decision‐making processes (Forester, 1984; Lindblom, 1959). For example, while risk ranks are determined annually and the immunization efforts will follow this ranking, surveillance budgets reported by PEI demonstrate an incremental increase over recent years that is not proportional to yearly case counts. EPP programmatic documents indicate that planned expenditures increase linearly across time‐periods within provinces.

In recent years, incidence, historic case counts, and immunity data have been utilized to construct predicted counts of future polio disease in upcoming years to try to improve ranking accuracy (Upfill‐Brown et al., 2014). Improved measurement of these indicators through enhanced surveillance could improve administrator understanding of where the risk of polio is greatest. Existing studies of surveillance have not attempted to link surveillance data to potential or lack‐of‐potential for changes in administrative allocation of funds: based on evidence from modeling however increased or targeted spending on surveillance can provide a necessary supplement that enhances the effectiveness of immunization actions (Thompson & Kalkowska, 2020). However, this return‐on‐investment is only achievable if surveillance efforts, whether using environmental samples or AFP, can support eradication decision making such that actual immunization activities are targeted to proper populations and tailored to the dynamics of the disease within those populations (Kalkowska, Duintjer Tebbens, & Thompson, 2019).

We evaluate this possibility using programmatic data from PEI and GPEI for the 2012 to 2016 (with 2017 case counts) period. Specifically, we develop a Bayesian decision analytic model which utilizes past administrative data as‐generated for provided predictive posteriors of the value of increased surveillance spending at the district level. In what follows, we detail the methodology, provide results and interpretations, and conclude by suggesting how surveillance budgetary allocations could best be targeted to enhance polio eradication efforts.

## 3 METHODS

We apply decision analysis and value of information (VOI) techniques to estimate the gains associated with incorporating additional surveillance information into models of disease presence and spread. Conducting a decision analysis leverages existing knowledge and data to explore future scenarios as well as the value of incorporating new information into a prospective decision (Heckerman, Geiger, & Chickering, 1995; Morgan, Henrion, & Small, 1992).

We utilize observations of six years of administrative rank data and disease indicators and use these data to develop model likelihoods for each branch in the decision tree. Because the risk of polio in any given district is dependent on the priors in each node, we analyze the risk rank decision as a Bayesian decision network which represents the decision as a Directed Acyclic Graph. The graph edges or connections are the probabilistic relationships associated with the variables fit via linear models, while each node is represented by the prior probability conditional on the values of the edge connections (Neapolitan et al., 2016). Because of the conditional independence assumption, we assume network connections between variables do not create dependency loops. This is a similar assumption to the classic conditional independence assumption utilized in decision trees or other decision analytic approaches.

This model is applicable for estimating the value of information because it allows the decisionmaker to set parameters and estimate the change in probability from adding new information or identifying risk more accurately within the model. A strength of using the Bayesian decision approach is that existing administrative data and expert knowledge of a system are used to establish a model of decision making and event probabilities (Heckerman et al., 1995). Specifically, we develop prior probabilities based on existing administrative data, and simulate posteriors based on observed associations. A weakness is that the model takes data as given to extrapolate values and returns.

The heart of value of information analysis is to exploit Bayesian updating to link data collection opportunities to likelihoods that represent both the nature of potentially obtainable data and also the structure of those data in light of the true “state of the world.” In other words, since most information is imperfect it is necessary to predict the true status of disease risk and probability of outbreak by district through manipulation and modeling informed by obtainable empirical measurements. It is essential to represent sources of uncertainty and variability to characterize distributed possible outcomes, as well as the tradeoffs that introduce tension for decision makers who must act without full knowledge of the future. The potential to resolve this opportunity loss is what confers a potential expected value on new information. By simulating a potential decision situation, we provide key evidence about how improved surveillance data can inform not only statistical models of disease outbreak and transmission but reduce uncertainty about relative priorities and risk ranks for policymakers.

**Fig 1:**
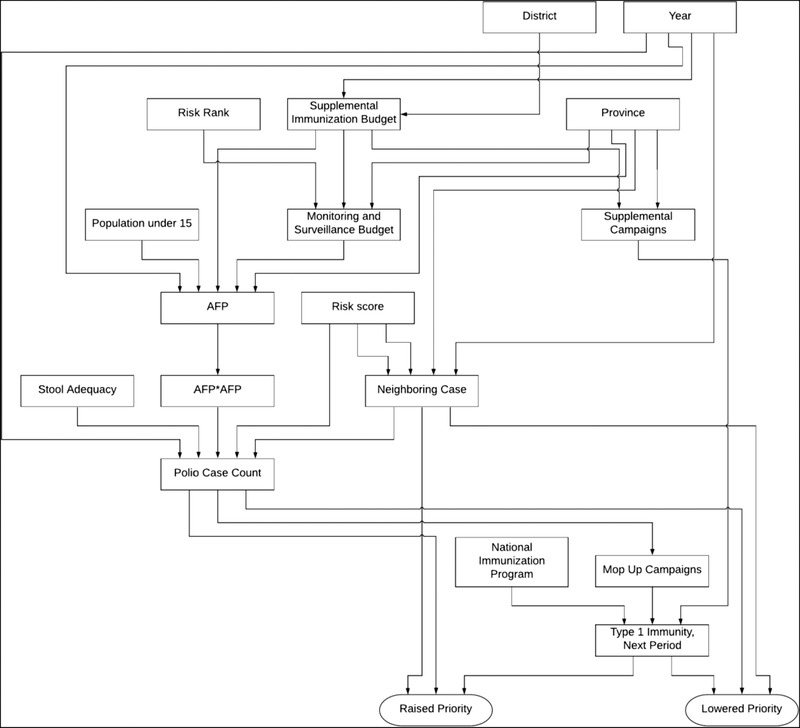
Influence diagram for model of decision regarding vaccination versus surveillance.

To develop a model of the decision making process, we reviewed primary source programmatic documents describing polio decision frameworks and targeting programs conducted by the World Health Organization and the Pakistan Polio Eradication Program, and gathered data on a variety of indicators related to surveillance and eradicating polio for the period from 2012–2016^3^. We used this document review to develop a descriptive framework representing the Pakistan Polio Eradication Program tier ranking process, with the goal of identifying programmatic variables and risk variables most commonly cited in describing the relative risks of each subarea. Using this framework, we construct an influence diagram representing our best understanding of how PEI assigns a risk ranking for subareas (Fig. 1).

### 3.1 Hybrid Bayesian Network Approach

Hybrid Bayesian networks include discrete and continuous nodes, which allow construction of a model with which probabilities can be updated as conditional on previously observed variables. In this case, preexisting administrative data from Pakistan are used to generate the Bayesian modeling structure above, such that the likelihood at each node is a function of all previous nodes. Because the underlying model is directed and acyclic, we cannot model loops or feedbacks between time periods, but we are able to represent how knowledge and outcomes in one time period influence disease planning inputs for the next. Accordingly, each district–year represents an observation where the estimates of the final nodes for that observation are shared as the input nodes for the next observation, such that each observation includes variables *t* and *t* + 1.

We use the R program HydeNet and the JAGS modeling framework to build a decision network that uses the collective administrative data to generate distributions for simulating posteriors under different surveillance scenarios (Nutter, n.d.; Plummer, 2004). For each district and year, we model surveillance spending as an outcome of the administrative risk rank. AFP case detection efforts and environmental surveillance are a function of this spending, which, informs detection of AFP, detection of polio positive cases of AFP, and finally, the administrative reranking of districts. For modeling, we fit a likelihood for each node or variable based on a linear, Poisson, or logit model depending on the variable. Posterior distributions are produced by simulating the network with prior likelihoods for each node to generate “new” cases or predictions based on the data as given. As a result, the posterior distribution at each node is conditional on the observations for all previous nodes (Fig. 1). Posterior distributions of the model are drawn via Monte Carlo simulation, with each draw representing a row in the data set or a district–year observation. We use twenty Monte Carlo chains with 10,000 iterations each to fit the model.

### 3.2 Data

**Fig 2:**
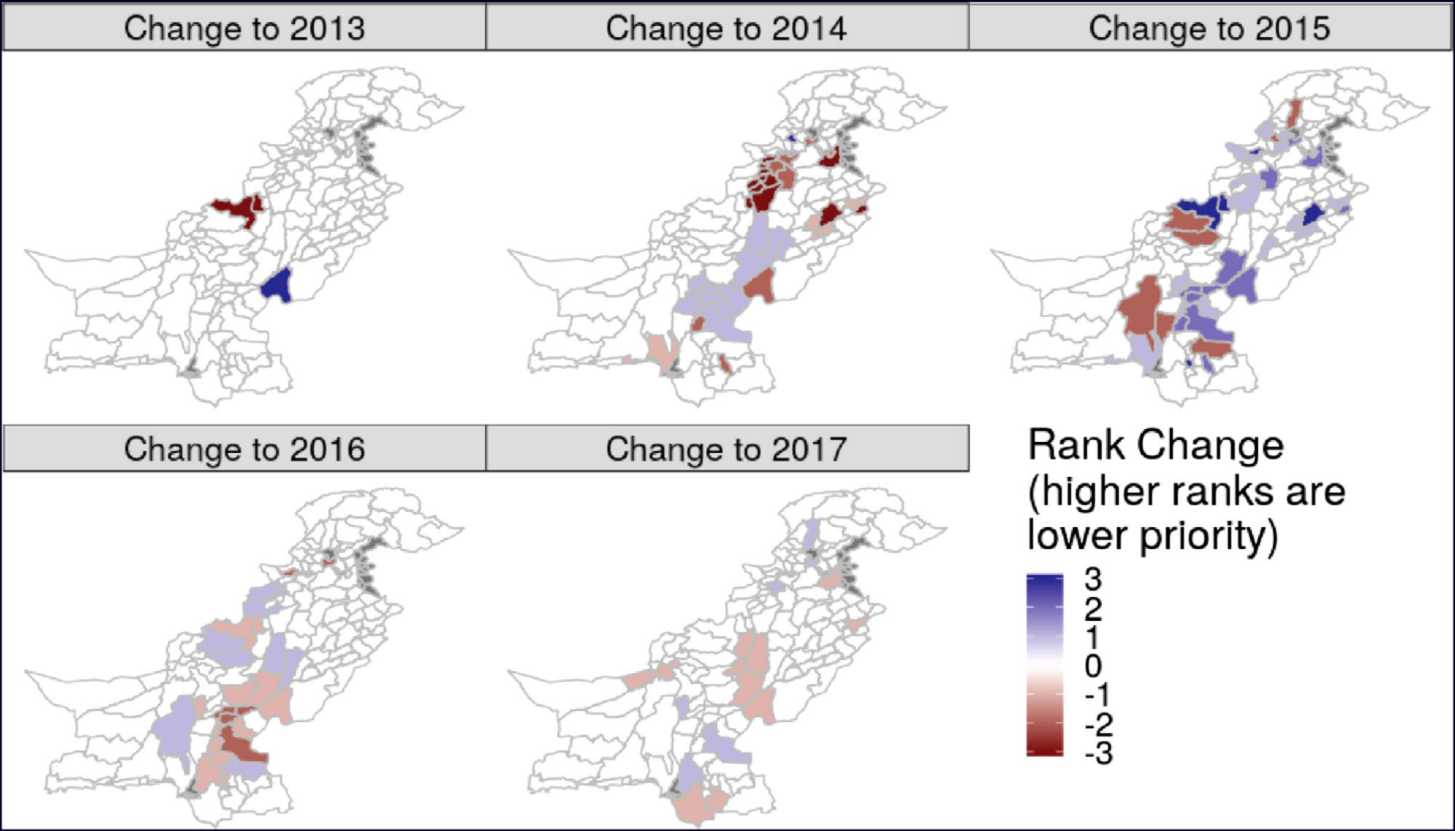
Change in polio priority year to year. A positive number is a decrease in priority while a negative number is an increase in priority, with 1 being highest priority and 4 being lowest priority.

We developed a comprehensive administrative data set by reviewing administrative risk ranks assigned to individual districts in Pakistan by the GPEI and the Pakistan emergency operations center—sources for our data are in Table I. Because the ranking method can be adjusted from year to year, we first synthesize the ranks by aligning rank criteria across years into four categories, with tier four representing the lowest risk, and tier one the highest risk. Between 2012 and 2017, changes relative to the previous year, that is, relative to the previous time step, are presented in Fig. 2.

**Table I:** Model Inputs, Units, and Source Information

Measure	Source	Unit
Risk rank (programmatic)	Pakistan polio eradication program annual/biannual reports, global polio eradication initiative reports/updates^1^	1:4 risk rank, with 1 being highest likelihood of disease
Expected Polio count	Institute for disease modeling (modeled internal result)	Predicted count of polio cases based on disease modeling
Surveillance and monitoring spending	Enhanced program on immunization—Pakistan documents^2^	USD
Supplementary immunization campaign vaccinations	Global polio eradication initiative^3^	Total vaccinations per district
Supplemental immunization budget by district	Enhanced program on immunization—Pakistan documents	USD
Population	World Bank under 15 population	Persons
AFP counts	Global polio eradication initiative	Count
National immunization campaign vaccinations	Global polio eradication initiative	Total vaccinations per district
Type 1 immunity	Global polio eradication initiative	%
Polio case count	Global polio eradication initiative	Count per district
Stool adequacy	Global polio eradication initiative	%
Raise priority	Pakistan polio eradication program annual/biannual reports, global polio eradication initiative reports/updates^4^	Binomial, calculated based on annual change
Lower priority	Pakistan polio eradication program annual/biannual reports, global polio eradication initiative reports/updates	Binomial, calculated based on annual change

^1^ See appendix for annual risk by district.

^2^ See appendix for annual spending by province.

^3^ GPEI data is proprietary and not publicly available.

^4^ See appendix for risk rankings.

We observe that changes in administrative rank are relatively rare and only occur in a minority of districts. As illustrated in Fig. 2, in total, there were 55 risk reductions and 69 risk increases out of 271 observations. Most districts are stable in high risk or low risk categories—for these districts, increased disease surveillance is unlikely to improve targeting of resources unless it later emerges that the original risk ranking was incorrect or priorities change. However, focusing only on those districts where change in rank occurs would result in a low overall sample size for the analysis, and ignores the fact that in future scenarios or years, rankings in these districts could change.

An important aspect of risk ranking is that the highest risk districts and the lowest risk districts present a challenge in that the ranks are bounded. Districts cannot surpass the highest risk category nor drop below the lowest risk category. We therefore determined that it was useful to consider rank changes based not only on whether districts move between ranks, but whether highest and lowest risk districts remained in such categories. We therefore classified rank increases and decreases into five categorical outcomes: “increase priority,” “decrease priority,” “same priority,” “stay highest priority,” and “stay lowest priority,” though, in this analysis we primarily focus on increasing and decreasing priority.

**Fig 3:**
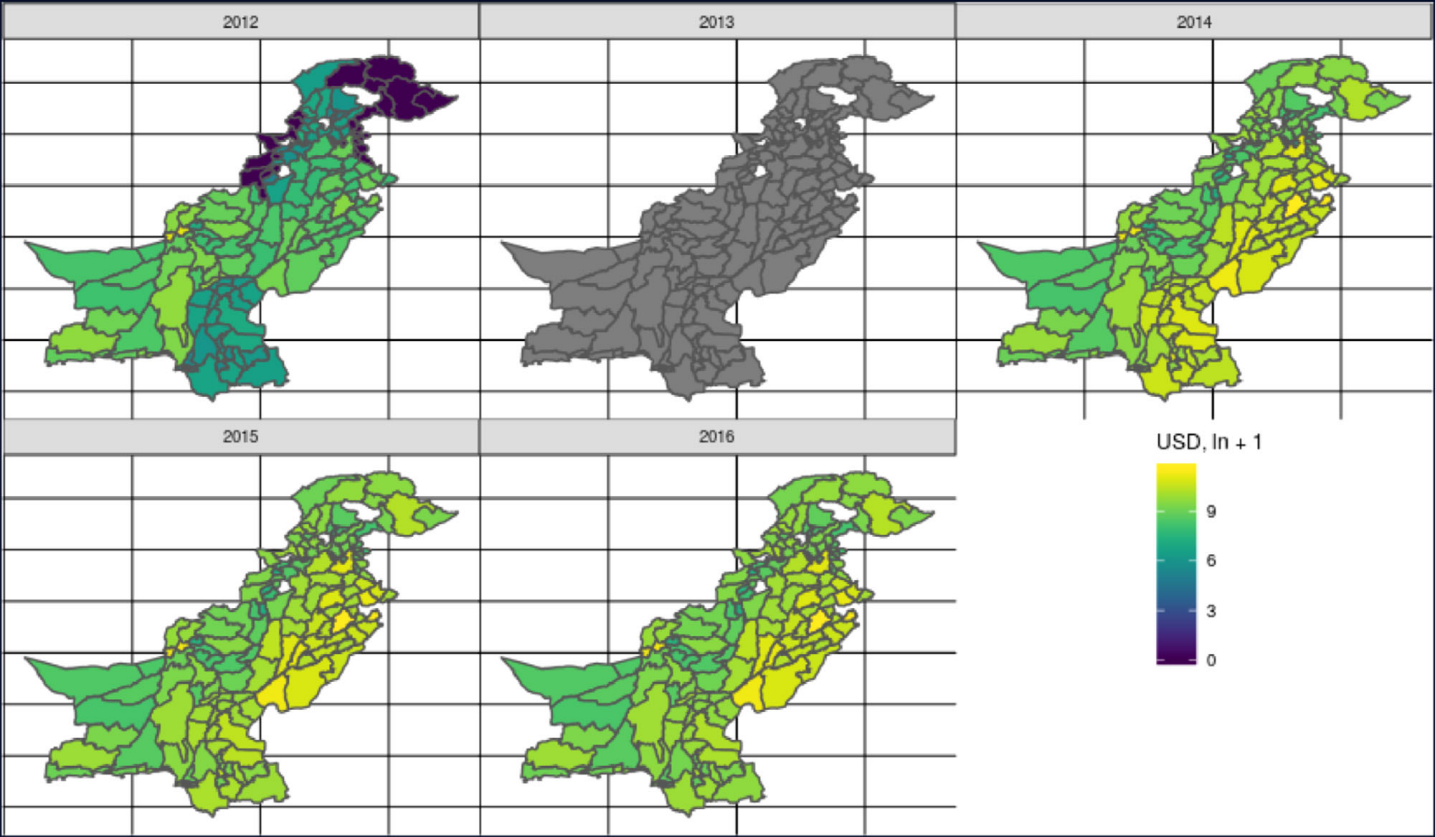
District surveillance spending in USD reported in provincial planning documents, scaling by district population. Color gradients are in US dollars on a ln scale.

Fig. 3 shows spending trends across Pakistan allocated from provinces to districts based on population. The budgets for 2012–2017 describe funding at the provincial level. We allocate funding from the surveillance budget to districts in proportion to the under‐15 years of age province population estimates from 2015. Notably, most districts have fairly consistent spending trends across years—not surprising given the potential for incremental budgetary processes and the allocation of provincial spending to districts based on population in the model (Berry, 1990; Guragain & Lim 2019). Median spending on polio surveillance within the data set for years with specific allocations for that purpose was $8,521 per district, or just $0.04 per person under‐15 per year. This is compared to an estimated $5.05 per person under‐5 per year spent on polio immunization campaigns. Thus, based on the data, even doubling spending on surveillance would contribute to a rather minor increase in annual polio eradication costs with budgeted surveillance allocations representing an average of less than 10% of immunization campaign costs at the provincial level. Our objective here is not to displace immunization spending, but instead, to point out that small increases in surveillance spending, if they increased efficacy in dispersing immunizations, could be a worthwhile investment for eradication of polio in Pakistan.

Because of the modeling framework, complete cases can be used at each node, meaning that for each modeled relationship in Fig. 1, any complete set of district–year data points for each node are used to represent the posterior. Thus, where funding information is not available, a district's AFP cases can still be utilized towards predicting polio case counts. This is critical as missing observations are a constant challenge in the data. Table II provides details of data gathered prior to modeling, demonstrating sample sizes and descriptives per province.

**Table II:** Basic Descriptives by Province

	Balochistan			Fata			Islamabad		
*Variable*	*N*	*Mean*	*SD*	*N*	Mean	*SD*	*N*	*Mean*	*SD*
Monitoring and surveillance spending	52	1.71E+04	1.75E+04	48	6.80E+03	7.42E+03	4	3.00E+03	2.71E+03
Supplemental immunization spending	52	3.90E+05	3.97E+05	48	2.96E+05	3.05E+05	4	3.87E+04	2.41E+04
AFP cases in district	65	1.29E+01	1.15E+01	60	2.74E+01	3.72E+01	5	1.66E+01	1.05E+01
Under 15 pop. of district	65	1.60E+05	1.62E+05	60	1.32E+05	1.05E+05	5	1.19E+05	0.00E+00
Count of AFP identified as polio	65	8.50E‐01	2.57E+00	60	6.77E+00	2.00E+01	5	0.00E+00	0.00E+00
Stool adequacy	65	8.60E‐01	1.70E‐01	54	8.10E‐01	1.30E‐01	5	9.00E‐01	7.00E‐02
Predicted count of polio cases	65	7.50E‐01	1.27E+00	60	4.78E+00	9.72E+00	5	1.00E‐01	3.00E‐02
Neighbor case	65	7.20E‐01	4.50E‐01	60	9.50E‐01	2.20E‐01	5	0.00E+00	0.00E+00
Supplemental campaigns	65	8.16E+01	1.48E+02	60	3.43E+01	8.39E+01	5	0.00E+00	0.00E+00
Mop up campaigns	65	5.10E‐01	1.40E+00	60	1.05E+00	5.79E+00	5	0.00E+00	0.00E+00
Immunity next period	52	5.80E‐01	7.00E‐02	48	6.40E‐01	1.90E‐01	4	7.60E‐01	7.00E‐02
National campaigns	65	0.00E+00	0.00E+00	60	4.18E+01	1.60E+02	5	0.00E+00	0.00E+00
Lowered priority (0,1)	52	0.19	0.40	48	0.12	0.33	4	0.25	0.50
Raised priority (0,1)	52	0.19	0.40	48	0.23	0.42	4	0.25	0.50
Same priority (0,1)	52	0.04	0.19	48	0.17	0.38	4	0.25	0.50

	*Sindh*			*KP*			*Punjab*		
*Variable*	*N*	*Mean*	*SD*	*N*	*Mean*	*SD*	*N*	*Mean*	*SD*
Monitoring and surveillance spending	60	6.98E+03	6.32E+03	60	6.98E+03	6.32E+03	48	4.51E+04	3.26E+04
Supplemental immunization spending	60	1.01E+06	8.50E+05	60	1.01E+06	8.50E+05	48	2.03E+06	1.02E+06
AFP cases in district	75	5.67E+01	3.89E+01	75	5.67E+01	3.89E+01	60	1.12E+02	5.39E+01
Under 15 pop. of district	75	4.06E+05	2.58E+05	75	4.06E+05	2.58E+05	60	1.42E+06	6.81E+05
Count of AFP identified as polio	75	1.96E+00	4.69E+00	75	1.96E+00	4.69E+00	60	2.20E‐01	6.10E‐01
Stool adequacy	75	8.00E‐01	1.30E‐01	75	8.00E‐01	1.30E‐01	60	8.80E‐01	4.00E‐02
Predicted count of polio cases	75	1.01E+00	1.38E+00	75	1.01E+00	1.38E+00	60	3.10E‐01	1.30E‐01
Neighbor case	75	9.20E‐01	2.70E‐01	75	9.20E‐01	2.70E‐01	60	4.70E‐01	5.00E‐01
Supplemental campaigns	75	2.21E+02	2.65E+02	75	2.21E+02	2.65E+02	60	4.52E+02	6.13E+02
Mop up campaigns	75	2.87E+00	1.10E+01	75	2.87E+00	1.10E+01	60	2.49E+00	1.22E+01
Immunity next period	60	7.60E‐01	7.00E‐02	60	7.60E‐01	7.00E‐02	48	7.80E‐01	4.00E‐02
National campaigns	75	0.00E+00	0.00E+00	75	0.00E+00	0.00E+00	60	0.00E+00	0.00E+00
Lowered priority (0,1)	60	0.22	0.42	60	0.22	0.42	48	0.21	0.41
Raised priority (0,1)	60	0.20	0.40	60	0.20	0.40	48	0.35	0.48
Same priority (0,1)	60	0.18	0.39	60	0.18	0.39	48	0.10	0.31

While the data gathered and illustrated in Table II are complete for most observations in the data set, there are notable gaps especially in terms of funding.

For example, disease surveillance spending is missing for 2013. Moreover, we can only observe changing in ranks for two‐year periods meaning that variable is missing for 2012. Thus, the hybrid Bayesian approach of using complete observations at each node allows us to maximize the information provided by existing data, given incompleteness. Here, we do not include missing data in computing priors but instead fit each node independently based on available data. The available data from 2013 can be used to inform the link between immunization campaigns and immunity, even if 2013 cannot inform the link between funding and campaigns.

## 4 RESULTS

We present results that allow all inputs to vary, generating correlations in posteriors across variables. Using these as a baseline we then compare scenarios with low and high spending on polio surveillance to illustrate the value of improved surveillance information for risk rank changes. We focus on three primary outcomes of interest. First, we evaluate generation of AFP cases, where AFP cases serve as the main indicator of quality poliovirus surveillance. It can be commonly assumed that all else held equal, increasing per‐district spending on surveillance should improve surveillance quality, which might then generate an increase in identification of AFP. Second, we evaluate how variables are associated with detection of new polio cases. AFP is only loosely associated with identification of polio cases. Therefore, a key limitation of surveillance is the link between AFP and polio cases.

Third, we evaluate posteriors for administrative risk rankings and rerankings. Essentially, we quantify the value of information via its potential to improve the decision about resource allocation made in time *t*+1. Efficacious spending on surveillance should improve prediction about where resources should be allocated to address polio risk in the next time period.

Table III presents model priors for nodes. Priors are estimated using likelihoods indicated in Table III. While each prior is fit with over 200 complete observations, there is still significant uncertainty in the relationship between observed variables and outcomes at various nodes—the uncertainty surrounding priors remains important—uncertainty for key inputs is included in the Supporting Information Appendix. We note that for key variables such as AFP cases, the relationship between spending and outcomes is not necessarily linear. Moreover, in many instances, variables present opposite relationships to expectations. For example, on average, identifying polio in a neighboring district is associated with a district being reclassified to a lower priority rank in the next time period—from a modeling perspective, districts in the low‐risk category are quite unlikely to increase in their risk rank, constraining the possible range of the coefficient. Essentially, at‐risk‐districts have the most potential to become lower risk districts.

**Table III:** Model Mean Coefficients and Standard Errors for Priors. Coefficients Represent Prior Predictions of How a One Unit Change in Each Variable Will Change the Outcome Variable

Outcome (*y*)	Transformation, model, intercept, Tau (1/variance^2^)	Variable (*x*)	Coefficient*
Year	Categorical, Uniform probability for each year	Simulated only based on distribution observed in data.	
Qualitative rank, start, 0:4	(0–4) Mean centered, normal,0,1		
District population	Ln, normal, 12.562,0.73022		
Province	Categorical, Uniform probability for each province		
Predicted cases	Ln, normal, −0.868, 0.707		
Stool adequacy, %	Normal, 0.85, 66.6		
National immunizations, count (NIA)	Normal, −4.327, 0.035		
Monitoring and surveillance spending (MSV) $	Ln, Normal, −6.487, 0.667	Qualitative Rank	0.27221
		Year	Varies
		SIAV	0.997
Supplemental immunization spending (SIAV) $	Ln, Normal, 13.575,11.233	District	Fixed effect
Neighboring case (0,1)	Logit, Bernoulli, 1.682	Risk Score	0.851
		Province	Varies
		Year	Varies
AFP cases in district (AFP)	Ln, Normal, −294.73,0.034	Under 15 Pop. of district	19.905
		MSV	1.691
		SIAV	4.541
Polio case count	Poisson, −18.548	Stool adequacy	−0.313
		AFP	0.0175
		AFP*AFP (AFP squared)	−6E‐05
		Predicted cases	0.851
		Province	Varies
		Neighbor case	18.25
Type 1 Immunity next period	Normal, 0.680, 8.66	Mop up campaigns	−0.00004
		SIA	0.00012
		National campaigns	−0.00024
Supplemental campaigns, count immunizations (SIA)	Ln, normal, −1535.780, 0.0033	SIAV	128.182
		Year	Varies
Mop up campaigns, count immunizations	Ln, normal, 2.8946, 0.097	Polio case count	0.212
Lowered priority	Binomial	Polio case count	−0.131
		Neighbor case	1.022
		Immunity next period	−0.971
Raised priority	Binomial	Polio case count	−0.128
		Neighbor case	0.206
		Immunity next period	2.781

### 4.1 Expectations of Increasing Average Polio Surveillance Budget by $100,000

Using the prior estimates, we simulate posterior observations of outcomes of interest, primarily, AFP cases, polio cases, and changes to the rank of districts under increased no‐surveillance spending alternatives. We used 20 chains of 10,000 simulations each for the no‐surveillance and increased surveillance alternatives, generating 40 models in total (20 for each spending condition).

**Fig 4:**
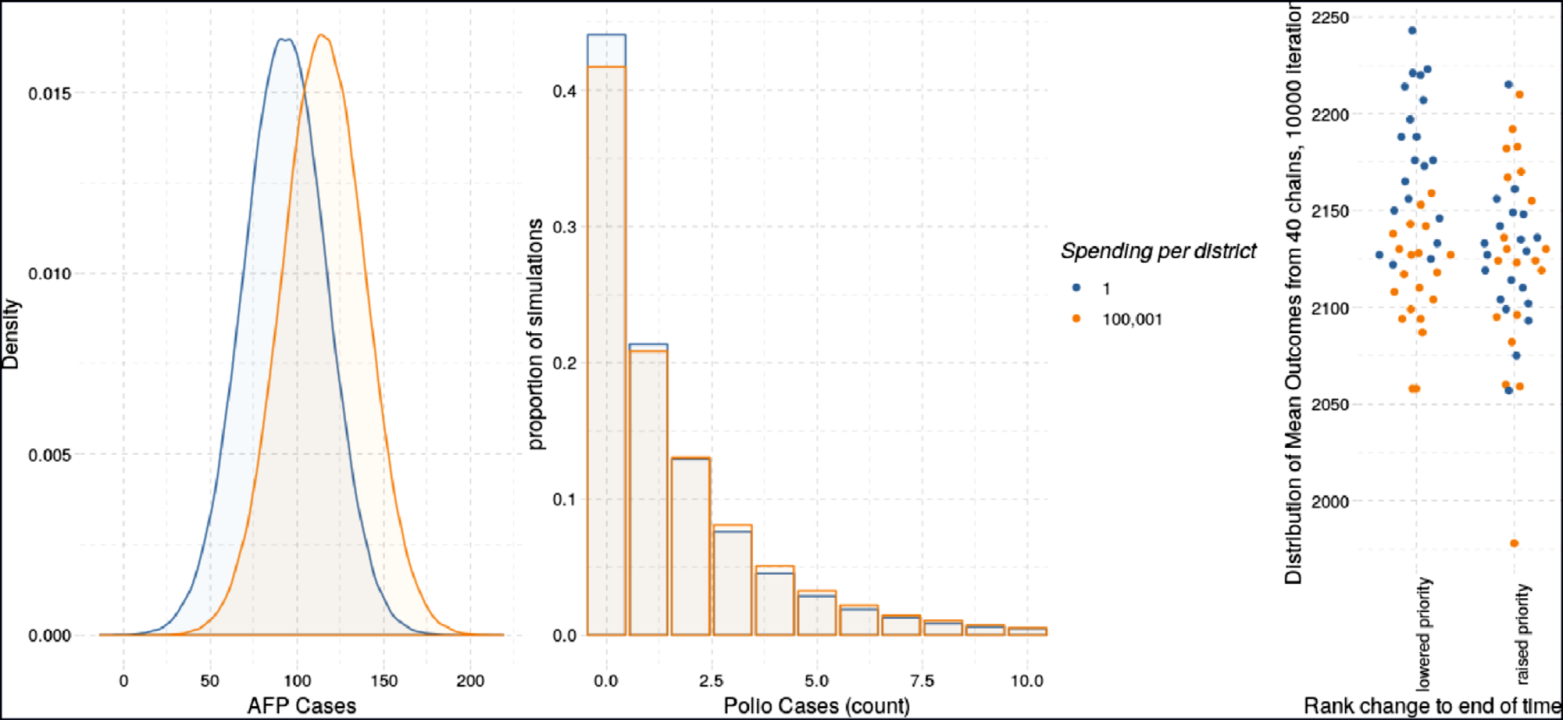
AFP case counts (panel 1), polio case counts (panel 2), and counts of observed changes to risk ranks at the end of the time period (panel 3).

Panel 1 of Fig. 4 describes increases in AFP as a function of a $100,000 increase in surveillance spending for that district. We use $100,000 here as a value because it represents a dramatic increase in surveillance spending: such an increase is unprecedented, and meant to highlight a maximal change to surveillance budgets rather than fitting within the small, incremental changes that regularly occur. In many districts, we observe less than $10,000 in spending. For these districts, $100,000 represents a 10‐fold increase. However, a $100,000 per district increase is equivalent to just over a 6.5% increase in the eradication budget nationally—adding just over $13,000,000 to the annual Polio budget. For context, annual GPEI outlays are generally over $200,000,000 for Pakistan alone. Thus, we selected $100,000 per district as an extreme case of increased spending in an attempt to identify possible returns on investment, recognizing actual spending increases in the field may be less; however, significant changes to occur in budgets at the provincial level so we do not believe this increase is unreasonable. For example, from 2012 to 2014, surveillance allocations in Sindh expanded from $22,500 to just over $1,100,000 for the 23 districts—an increase of just under $50,000 averaged across all districts. The next year allocations returned to approximately $677,000 (2015). Monitoring and surveillance spending in the federal territory also regularly varied in our data set by over $1,000,000, indicating changes of $100,000 per district are extreme but not unreasonable.

As expected, given priors, spending on surveillance is associated with increased identification of AFP cases. On average, a $100,000 increase in surveillance spending per district is associated with an 11.5 count increase in the cases of AFP per district per year, holding all else equal.

Moving to panel 2 of Fig. 4, increased spending on surveillance is associated with increased identification of polio cases, as indicated by fewer zero‐count districts. However, the change in the proportion of districts with zero cases is small, less than 3%. The small difference in the distribution of cases in panel 2 relative to the change in AFP in panel 1 is indicative of the limited ability of AFP to detect polio—relying on AFP cases for detection fundamentally limits how many cases will be caught (1/200). This propagates to panel 3 of Fig. 4.

Panel 3 depicts whether districts increased or decreased in priority ranking, with the count of districts experiencing each change in the 40 model simulations presented on the *y*‐axis. While increased spending simulations are likely to have a smaller number of districts lowering their priority ranking, there is no difference in the number of districts raising their priority.

**Fig 5:**
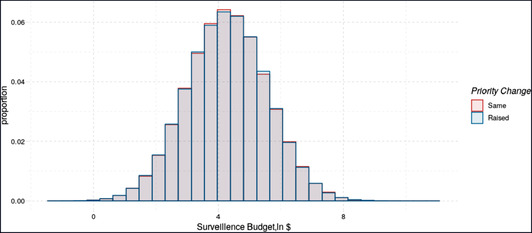
Distribution of the surveillance budget and priority rank changes. There is some evidence that higher surveillance budgets overall are associated with increased priority raising, but, the distribution of outcomes is relatively similar across surveillance budgets.

However, the value of information approach is based on the ability to observe posteriors under different scenarios. We therefore also simulated two conditions—districts that had an increase in rank and districts that had a decrease in rank, with the goal of seeing how the distribution of spending might differ given a rank change. Fig. 5 presents surveillance budgets on the *x*‐axis and priority rank changes for 400,000 simulated districts, comparing districts that increased in priority to those that stayed the same. Here, we observe that as budgeted spending on surveillance increases, there is a marginal shift toward raising the priority of a district versus maintaining it at the same priority, but, the shift is relatively small.

**Fig 6:**
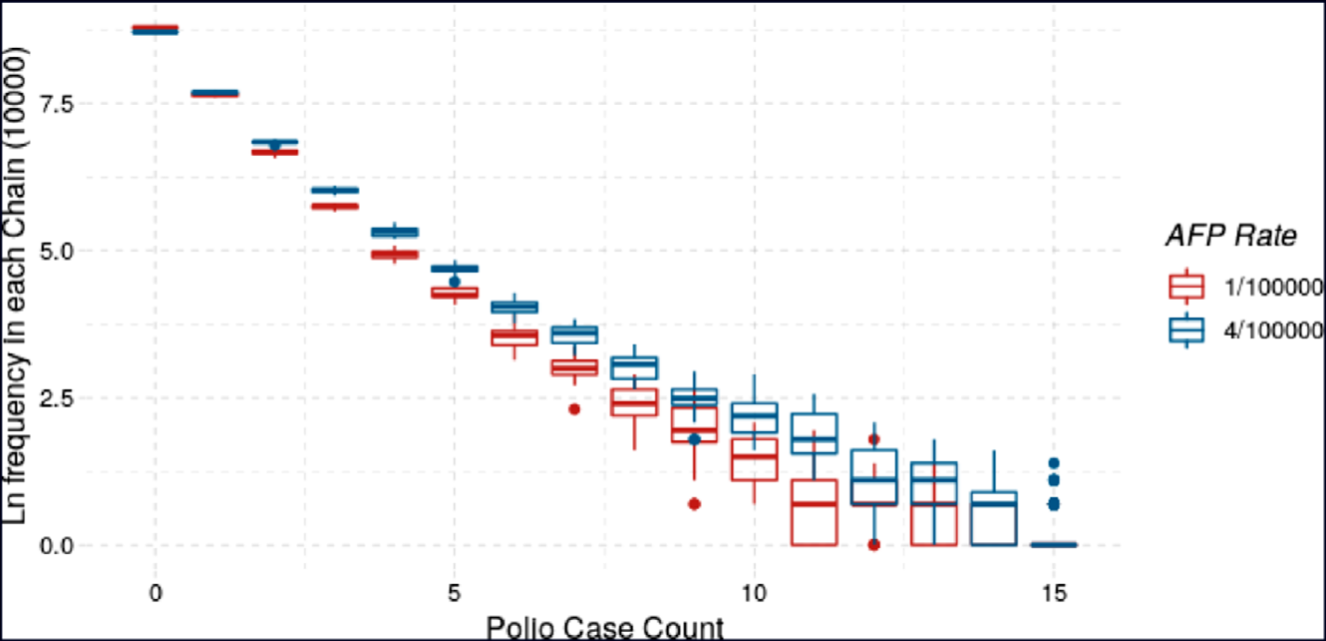
Frequency (ln) of identifying given counts of polio cases in each district at different AFP values. Higher AFP rates do tend to identify more polio cases given the data in Pakistan, that is: presence of polio in districts is a requirement for this finding.

In Fig. 6, our model suggests that increasing existing polio surveillance efforts has different effects for high and low risk districts. Increasing AFP detection rate will identify low‐risk areas that should become high risk only rarely as the probability of finding new polio cases is low, whereas high‐risk areas are likely to stay high risk with increased spending on surveillance.

**Fig 7:**
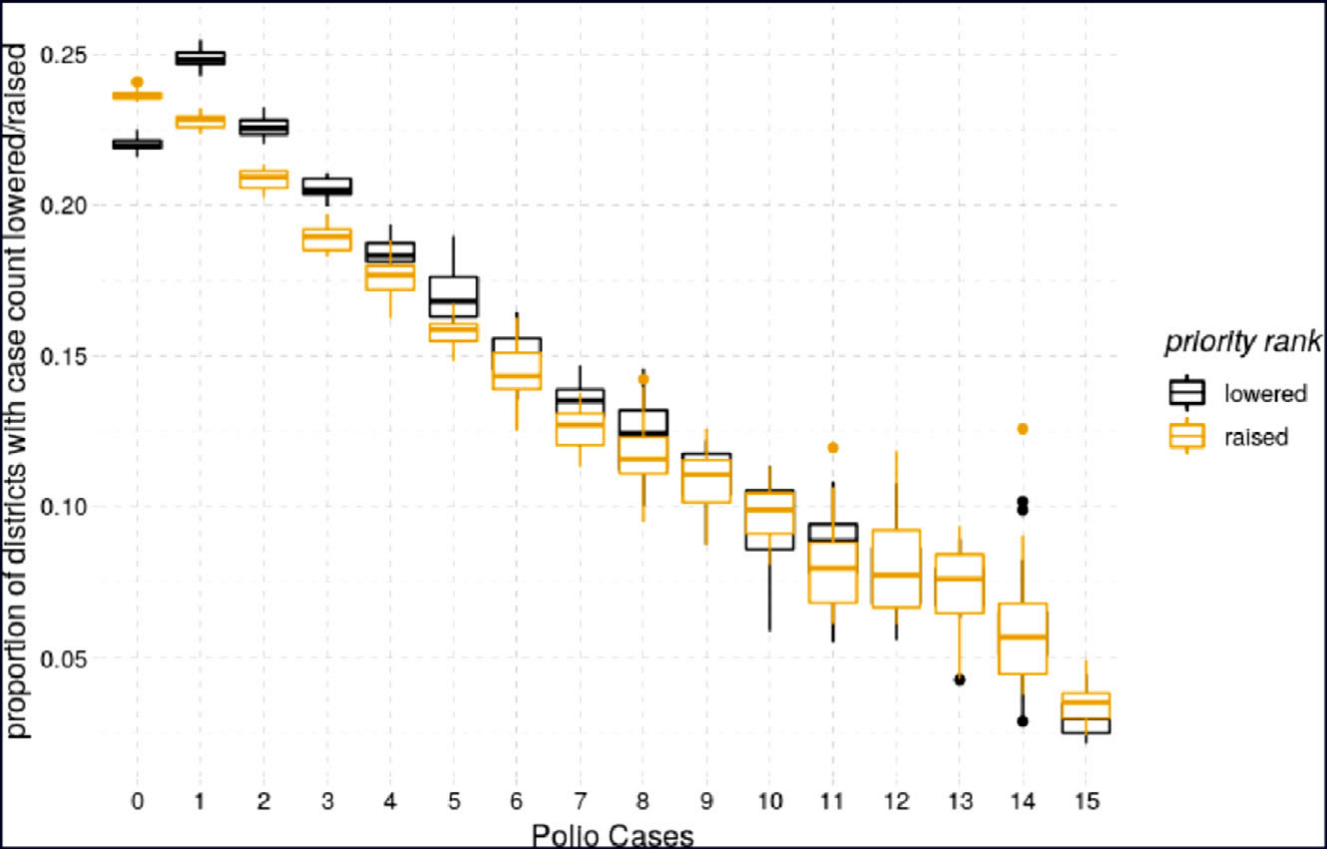
As more cases are identified in a simulated year, the likelihood of raising the rank increases relative to the risk lowering which occurs at low counts; however, there is only a difference between the two distributions at low case counts.

In Fig. 7, we explicitly compare the impact of the number of cases detected on the probability of the priority rank being raised or lower, we see that the greatest difference happens when 0, 1, 2, or 3 cases are detected. When the number of cases detected is greater, increasing or decreasing the AFP rate is unlikely to change the priority rank. Thus, improving surveillance is most impactful in areas with poor surveillance and/or small number of detected cases. Historically, few districts have polio cases—in our sample for example, 80 districts never had a single case of polio. Districts that change risk ranking are most often those that have had at least one polio case in the time period, meaning immunization efforts are already likely to occur there.

## 5 DISCUSSION AND CONCLUSION

We acknowledge that AFP surveillance has only limited ability to detect poliovirus circulation—this is because few polio cases result in AFP. At best only 1 per 200 polio infections is detectable as AFP. Thus, improving AFP surveillance alone has a limited ability to change immunization campaign planning because of how risk rankings interact with polio incidence, even though AFP surveillance serves to illuminate polio disease dynamics and to better predict outbreaks.

The correlation between surveillance spending and reduced risk of misclassification was limited. This could be because sufficient surveillance has been achieved in the key districts, and that additional efforts would not uncover additional cases and thus would not change the risk prioritization. The biggest changes in risk come from differentiating the absence of cases from low incidence. There are many districts with zero case counts, and only a small fraction of these are likely to become infected, which thus reduces the return on investment for increasing surveillance spending in districts with zero cases.

The polio program's efforts at broad coverage might thus result in low returns for surveillance efforts overall in terms of within‐country allocation even if it captures reemergence in previously low‐risk districts. Where the risk is highest, efforts to detect transmission can potentially uncover more cases, but, if the area was already at high risk, the benefit of this finding is limited as new knowledge cannot be introduced into the risk ranking of that area. Historically, reclassification of districts to increased risk‐rank primarily occurred in 2016—moreover, the districts that were reranked did not go from no risk to high risk, but rather from a medium risk category to a higher risk category, indicating even new cases mostly confirm expected outcomes.

In our model, funding additional AFP surveillance has limited impact on risk ranking. Changes in rank were most likely when additional surveillance funding was allocated to areas with no or few cases—potentially indicative of the benefit for surveillance in detecting unexpected outbreaks not prioritizing interventions in known risk areas. Further, a district rarely underwent a change in rank based on identification of polio in that district alone, while including neighboring polio cases as predictors was associated with a limited impact.

To achieve a more efficient resource allocation, one would have to exploit different sources of information to inform risk ranking. For example, ES is more sensitive than disease surveillance, thus adding environmental information when feasible will help. Furthermore, the PEI currently considers nondisease information during resource prioritization, for example, vaccination coverage monitoring, operational performance, and local social information. These measures are more difficult to quantify and relate to disease incidence; however, they too have the potential to inform prioritization beyond what our current model incorporates.

Analyzing expenditures in more detail (we relied on province‐level data in this analysis) could also suggest novel ways to optimize funding allocation so as to maximize the probability of eradication. Tracking how expenditures change in response to changing epidemiology, as well as how epidemiology responds to changing expenditures, would improve understanding of the relationship between polio eradication efforts and polio circulation, and would suggest where more funds would help and where funds could be reduced without increasing overall polio outbreak risk. While in the past, attention to financial monitoring has been limited, new technologies have tremendously improved the ability to trace returns to investment from each dollar spent. Platforms such as OpenGov have made documenting and sharing investment data in a transparent, consistent manner the norm for many public agencies although we acknowledge that this takes commitment and resources (OpenGov, n.d.). Expanding these transparency and tracking tools to additional areas of application such as public health program monitoring could enhance the ability to tie interventions to outcomes.

## FUNDING STATEMENT

This work was supported by the Bill & Melinda Gates Foundation [Institute for Disease Modeling]. Additionally, partial support for this research came from a Eunice Kennedy Shriver National Institute of Child Health and Human Development research infrastructure grant, P2C HD042828, to the Center for Studies in Demography & Ecology at the University of Washington.

## Supporting information


**Table A1**: Risk rankings by year.
**Table A2**: Budget Allocations by year.
**Table A3**: Model Fitting and Transformations

## Footnotes

1 Example: https://reliefweb.int/sites/reliefweb.int/files/resources/NEAP‐2020.pdf

2 Example: https://reliefweb.int/sites/reliefweb.int/files/resources/Final‐pakistan‐NEAP2015‐16.pdf, pg 29

3 Excluding 2014 because there is no funding data for that year. 2014 data is in our analysis in terms of lags of case data and AFP measures, so it informs some priors but not the link between spending and outcomes.
