# Supplementary material for: Disease Surveillance Investments and Administration: Limits to Information Value in Pakistan Polio Eradication
Source: Risk Anal. 2020 Aug 21;41(2):273–88. doi: 10.1111/risa.13580 (PMC7984073; doi:10.1111/risa.13580)
Supplement: Supplementary file 1 — Table A1: Risk rankings by year. Table A2: Budget Allocations by year. Table A3: Model Fitting and Transformations [file RISA-41-273-s001.docx]

# Appendix

Table A1: Risk rankings by year. Risk ranks are gathered from annual programmatic documents, specifically the National Emergency Action Plan documents for 2012-2017.

| PROVINCE | DISTRICT | 2012 | 2013 | 2014 | 2015 | 2016 | 2017 |
| --- | --- | --- | --- | --- | --- | --- | --- |
| Balochistan | Awaran | 4 | 4 | 4 | 4 | 4 | 4 |
| Balochistan | Barkhan | 4 | 4 | 4 | 4 | 4 | 3 |
| Balochistan | Chagai | 4 | 4 | 4 | 4 | 4 | 4 |
| Balochistan | Dera Bugti | 4 | 4 | 4 | 4 | 3 | 3 |
| Balochistan | Gwadar | 4 | 4 | 4 | 4 | 4 | 4 |
| Balochistan | Harnai | 4 | 4 | 4 | 4 | 4 | 4 |
| Balochistan | Jaffarabad | High | High | High-2 | 3 | 3 | 3 |
| Balochistan | Jhal Magsi | 4 | 4 | 4 | 4 | 3 | 4 |
| Balochistan | Kachhi | 4 | 4 | 4 | 4 | 4 | 4 |
| Balochistan | Kalat | 4 | 4 | 4 | 4 | 4 | 4 |
| Balochistan | Kech | 4 | 4 | 4 | 4 | 4 | 4 |
| Balochistan | Kharan | 4 | 4 | 4 | 4 | 4 | 4 |
| Balochistan | Khuzdar | 4 | 4 | 4 | 2 | 3 | 3 |
| Balochistan | Killa Abdullah | High | High | High-1 | High-1 | 1 | 1 |
| Balochistan | Killa Saifullah | 4 | 4 | 4 | 2 | 3 | 3 |
| Balochistan | Kohlu | 4 | 4 | 4 | 4 | 4 | 4 |
| Balochistan | Las Bela | 4 | 4 | 4 | 4 | 4 | 4 |
| Balochistan | Lehri | 4 | 4 | 4 | 4 | 4 | 4 |
| Balochistan | Loralai | 4 | 4 | 4 | 2 | 3 | 3 |
| Balochistan | Mastung | 4 | 4 | 4 | 4 | 4 | 3 |
| Balochistan | Musakhel | 4 | 4 | 4 | 4 | 4 | 3 |
| Balochistan | Nasirabad | High | High | High-2 | 3 | 3 | 3 |
| Balochistan | Nushki | 4 | 4 | 4 | 4 | 4 | 3 |
| Balochistan | Panjgur | 4 | 4 | 4 | 4 | 4 | 4 |
| Balochistan | Pishin | High | High | High-1 | High-1 | 1 | 1 |
| Balochistan | Quetta | High | High | High-1 | High-1 | 1 | 1 |
| Balochistan | Sheerani | 4 | High | 1 | 4 | 3 | 3 |
| Balochistan | Sibi | 4 | 4 | 4 | 4 | 4 | 4 |
| Balochistan | Sohbatpur | 4 | 4 | 4 | 4 | 4 | 4 |
| Balochistan | Washuk | 4 | 4 | 4 | 4 | 4 | 4 |
| Balochistan | Zhob | 4 | High | 1 | 4 | 3 | 3 |
| Balochistan | Ziarat | 4 | 4 | 4 | 4 | 4 | 4 |
| Fata | Bajaur Agency | High | High | High-1 | 2 | 2 | 2 |
| Fata | FR Bannu | 4 | 4 | 1 | 1 | 2 | 2 |
| Fata | FR Dera Ismail Khan | 4 | 4 | 1 | 2 | 2 | 2 |
| Fata | FR Kohat | 4 | 4 | 1 | 2 | 2 | 2 |
| Fata | FR Lakki Marwat | 4 | 4 | 1 | 2 | 2 | 2 |
| Fata | FR Peshawar | 4 | 4 | 1 | 2 | 2 | 2 |
| Fata | FR Tank | 4 | 4 | 1 | 2 | 2 | 2 |
| Fata | Khyber Agency | High | High | High-1 | High-1 | 1 | 1 |
| Fata | Kurram Agency | High | High | High-1 | 2 | 2 | 2 |
| Fata | Mohmand Agency | High | High | High-1 | 2 | 2 | 2 |
| Fata | North Waziristan Agency | High | High | High-1 | High-1 | 2 | 2 |
| Fata | Orakzai Agency | High | High | High-1 | 4 | 2 | 2 |
| Fata | South Waziristan Agency | High | High | High-1 | High-1 | 2 | 2 |
| Federal Capital Territory | Islamabad | 4 | 4 | 2 | 3 | 3 | 2 |
| Baltistan | Astore | 4 | 4 | 4 | 4 | 4 | 4 |
| Baltistan | Diamer | 4 | 4 | 4 | 4 | 4 | 4 |
| Baltistan | Ghanche | 4 | 4 | 4 | 4 | 4 | 4 |
| Baltistan | Ghizer | 4 | 4 | 4 | 4 | 4 | 4 |
| Baltistan | Gilgit | 4 | 4 | 4 | 4 | 4 | 4 |
| Baltistan | Hunza | 4 | 4 | 4 | 4 | 4 | 4 |
| Baltistan | Kharmang | 4 | 4 | 4 | 4 | 4 | 4 |
| Baltistan | Nagar | 4 | 4 | 4 | 4 | 4 | 4 |
| Baltistan | Shigar | 4 | 4 | 4 | 4 | 4 | 4 |
| Baltistan | Skardu | 4 | 4 | 4 | 4 | 4 | 4 |
| Khyber Pakhtunkhwa | Abbottabad | 4 | 4 | 4 | 4 | 4 | 4 |
| Khyber Pakhtunkhwa | Bannu | 4 | 4 | 1 | High-1 | 2 | 2 |
| Khyber Pakhtunkhwa | Batagram | 4 | 4 | 4 | 4 | 4 | 4 |
| Khyber Pakhtunkhwa | Buner | 4 | 4 | High-1 | 2 | 4 | 4 |
| Khyber Pakhtunkhwa | Charsadda | High | High | 4 | 2 | 2 | 2 |
| Khyber Pakhtunkhwa | Chitral | 4 | 4 | 4 | 4 | 4 | 4 |
| Khyber Pakhtunkhwa | Dera Ismail Khan | 4 | 4 | 1 | 2 | 2 | 2 |
| Khyber Pakhtunkhwa | Hangu | 4 | 4 | 1 | 2 | 2 | 2 |
| Khyber Pakhtunkhwa | Haripur | 4 | 4 | 4 | 4 | 4 | 4 |
| Khyber Pakhtunkhwa | Karak | 4 | 4 | 2 | 2 | 2 | 3 |
| Khyber Pakhtunkhwa | Kohat | 4 | 4 | 2 | 2 | 2 | 2 |
| Khyber Pakhtunkhwa | Kohistan | 4 | 4 | 4 | 4 | 4 | 4 |
| Khyber Pakhtunkhwa | Lakki Marwat | 4 | 4 | 1 | 2 | 2 | 2 |
| Khyber Pakhtunkhwa | Lower Dir | 4 | 4 | 4 | 4 | 4 | 4 |
| Khyber Pakhtunkhwa | Malakand PA | 4 | 4 | 4 | 4 | 4 | 4 |
| Khyber Pakhtunkhwa | Mansehra | 4 | 4 | 4 | 4 | 4 | 4 |
| Khyber Pakhtunkhwa | Mardan | High | High | High-1 | 2 | 2 | 2 |
| Khyber Pakhtunkhwa | Nowshera | High | High | High-1 | 2 | 2 | 2 |
| Khyber Pakhtunkhwa | Peshawar | High | High | High-1 | High-1 | 1 | 1 |
| Khyber Pakhtunkhwa | Shangla | 4 | 4 | 4 | 4 | 4 | 4 |
| Khyber Pakhtunkhwa | Swabi | 4 | 4 | 2 | 4 | 2 | 3 |
| Khyber Pakhtunkhwa | Swat | 4 | 4 | 4 | 2 | 2 | 3 |
| Khyber Pakhtunkhwa | Tank | 4 | 4 | 1 | High-1 | 2 | 2 |
| Khyber Pakhtunkhwa | Tor Ghar | 4 | 4 | 2 | 4 | 4 | 4 |
| Khyber Pakhtunkhwa | Upper Dir | 4 | 4 | 4 | 4 | 4 | 4 |
| Punjab | Attock | 4 | 4 | 4 | 4 | 4 | 4 |
| Punjab | Bahawalnagar | 4 | 4 | 4 | 4 | 4 | 4 |
| Punjab | Bahawalpur | 4 | 4 | 4 | 4 | 4 | 4 |
| Punjab | Bhakkar | 4 | 4 | 4 | 4 | 4 | 4 |
| Punjab | Chakwal | 4 | 4 | 4 | 4 | 4 | 4 |
| Punjab | Chiniot | 4 | 4 | 4 | 4 | 4 | 4 |
| Punjab | Dera Ghazi Khan | High | High | High-2 | 2 | 3 | 2 |
| Punjab | Faisalabad | 4 | 4 | 1 | 4 | 4 | 4 |
| Punjab | Gujranwala | 4 | 4 | 4 | 4 | 4 | 4 |
| Punjab | Gujrat | 4 | 4 | 4 | 4 | 4 | 4 |
| Punjab | Hafizabad | 4 | 4 | 4 | 4 | 4 | 4 |
| Punjab | Jhang | 4 | 4 | 4 | 4 | 4 | 4 |
| Punjab | Jhelum | 4 | 4 | 4 | 4 | 4 | 4 |
| Punjab | Kasur | 4 | 4 | 4 | 4 | 4 | 4 |
| Punjab | Khanewal | 4 | 4 | 4 | 4 | 4 | 4 |
| Punjab | Khushab | 4 | 4 | 4 | 4 | 4 | 4 |
| Punjab | Lahore | 4 | 4 | 1 | 3 | 3 | 3 |
| Punjab | Layyah | 4 | 4 | 4 | 4 | 4 | 4 |
| Punjab | Lodhran | 4 | 4 | 4 | 4 | 4 | 4 |
| Punjab | Mandi Bahauddin | 4 | 4 | 4 | 4 | 4 | 4 |
| Punjab | Mianwali | 4 | 4 | High-2 | 4 | 4 | 4 |
| Punjab | Multan | High | High | High-2 | 3 | 3 | 3 |
| Punjab | Muzaffargarh | High | High | High-2 | 2 | 3 | 3 |
| Punjab | Nankana Sahib | 4 | 4 | 4 | 4 | 4 | 4 |
| Punjab | Narowal | 4 | 4 | 4 | 4 | 4 | 4 |
| Punjab | Okara | 4 | 4 | 4 | 4 | 4 | 4 |
| Punjab | Pakpattan | 4 | 4 | 4 | 4 | 4 | 4 |
| Punjab | Rahim Yar Khan | High | 4 | High-2 | 4 | 3 | 2 |
| Punjab | Rajanpur | High | High | High-2 | 4 | 3 | 2 |
| Punjab | Rawalpindi | 4 | 4 | 1 | 3 | 3 | 2 |
| Punjab | Sahiwal | 4 | 4 | 4 | 4 | 4 | 4 |
| Punjab | Sargodha | 4 | 4 | 4 | 4 | 4 | 4 |
| Punjab | Sheikhupura | 4 | 4 | 4 | 4 | 4 | 3 |
| Punjab | Sialkot | 4 | 4 | 4 | 4 | 4 | 4 |
| Punjab | Toba Tek Singh | 4 | 4 | 4 | 4 | 4 | 4 |
| Punjab | Vehari | 4 | 4 | 4 | 4 | 4 | 4 |
| Sindh | Badin | 4 | 4 | 4 | 4 | 4 | 3 |
| Sindh | Dadu | 4 | 4 | 4 | 2 | 2 | 2 |
| Sindh | Ghotki | High | High | High-2 | 2 | 2 | 2 |
| Sindh | Hyderabad | High | High | High-1 | 4 | 3 | 3 |
| Sindh | Jacobabad | High | High | High-2 | 4 | 2 | 2 |
| Sindh | Jamshoro | 4 | 4 | 4 | 4 | 3 | 4 |
| Sindh | Karachi City | High | High | High-1 | High-1 | 1 | 1 |
| Sindh | Kashmore | High | High | High-2 | 4 | 2 | 2 |
| Sindh | Khairpur | High | High | High-2 | 4 | 2 | 3 |
| Sindh | Larkana | High | High | High-2 | 3 | 2 | 2 |
| Sindh | Matiari | 4 | 4 | 4 | 4 | 3 | 3 |
| Sindh | Mirpur Khas | 4 | 4 | 2 | 4 | 4 | 4 |
| Sindh | Naushahro Feroze | 4 | 4 | 2 | 4 | 3 | 3 |
| Sindh | Qambar Shahdadkot | High | High | High-2 | 2 | 2 | 2 |
| Sindh | Sanghar | 4 | 4 | 4 | 2 | 3 | 3 |
| Sindh | Shaheed Benazirabad | 4 | 4 | 4 | 4 | 3 | 3 |
| Sindh | Shikarpur | High | High | High-2 | 4 | 2 | 2 |
| Sindh | Sujawal | 4 | 4 | 4 | 4 | 4 | 3 |
| Sindh | Sukkur | High | High | High-2 | 3 | 2 | 2 |
| Sindh | Tando Allah Yar | 4 | 4 | 4 | 4 | 4 | 4 |
| Sindh | Tando Muhammad Khan | 4 | 4 | 4 | 4 | 4 | 4 |
| Sindh | Tharparkar | 4 | 4 | 4 | 4 | 4 | 4 |
| Sindh | Thatta | 4 | 4 | 4 | 4 | 4 | 3 |
| Sindh | Umerkot | 4 | 4 | 4 | 4 | 4 | 4 |

Table A2: Budget Allocations by year. Derived from Pakistan Comprehensive Multi-year Plans

|  |  | 2012 | 2014 | 2015 | 2016 | 2017 | 2018 |
| --- | --- | --- | --- | --- | --- | --- | --- |
| Surveillance and Monitoring | **BAL** | **282981.00** | **344250.00** | **358158.00** | **372624.00** | **387682.00** | **403342.00** |
| Detection and Notification | BAL | 127341.00 | 137700.00 | 143263.00 | 149051.00 | 155072.00 | 161337.00 |
| Case and outbreak verification and investigation | BAL | 99043.00 | 112200.00 | 116733.00 | 121449.00 | 126354.00 | 131460.00 |
| Data Management | BAL | 56597.00 | 86700.00 | 90203.00 | 93847.00 | 97639.00 | 101580.00 |
| Laboratory | BAL | 0.00 | 7650.00 | 7959.00 | 8277.00 | 8616.00 | 8965.00 |
| Supportive Activities | BAL | 0.00 | 0.00 | 0.00 | 0.00 | 0.00 | 0.00 |
| Surveillance and Monitoring | KP | 15555.55556 | 198965.6993 | 226752.5906 | 261917.3714 | 291614.4161 | 380584.7127 |
| Detection and Notification | KP | 0 | 12532.366 | 14065.93056 | 15781.96701 | 17707.36338 | 19867.6519 |
| Case and outbreak verification and investigation | KP | 9444.444444 | 34566.66667 | 36414 | 40874.1948 | 45841.00198 | 51433.60422 |
| Data Management | KP | 6111.111111 | 37966.66667 | 41714.26 | 50879.028 | 58890.32302 | 66197.61807 |
| Laboratory | KP | 0 | 8500 | 13872 | 17686.8 | 21648.6432 | 24535.12896 |
| Supportive Activities | KP | 0 | 105400 | 120686.4 | 136695.3816 | 147527.0845 | 218550.7095 |
| Surveillance and Monitoring | GB | 0 | 111180 | 115671.672 | 120344.1708 | 125204.9279 | 130262.7654 |
| Detection and Notification | GB | 0 | 45900 | 47754.36 | 49683.63614 | 51690.46537 | 53778.67184 |
| Case and outbreak verification and investigation | GB | 0 | 36720 | 38203.488 | 39746.48443 | 41352.15581 | 43022.71666 |
| Data Management | GB | 0 | 28560 | 29713.824 | 30914.05025 | 32162.30677 | 33461.3769 |
| Laboratory | GB | 0 | 0 | 0 | 0 | 0 | 0 |
| Supportive Activities | GB | 0 | 0 | 0 | 0 | 0 | 0 |
| Surveillance and Monitoring | AJK | 0 | 76500 | 79590.6 | 82806.06024 | 86151.42507 | 89631.94265 |
| Detection and Notification | AJK | 0 | 30600 | 31836.24 | 33122.4241 | 34460.57003 | 35852.77706 |
| Case and outbreak verification and investigation | AJK | 0 | 25500 | 26530.2 | 27602.02008 | 28717.14169 | 29877.31422 |
| Data Management | AJK | 0 | 20400 | 21224.16 | 22081.61606 | 22973.71335 | 23901.85137 |
| Laboratory | AJK | 0 | 0 | 0 | 0 | 0 | 0 |
| Supportive Activities | AJK | 0 | 0 | 0 | 0 | 0 | 0 |
| Surveillance and Monitoring | PUN | 196148 | 1473535.86 | 1531877.677 | 1531912.114 | 1659365.99 | 1658190.939 |
| Detection and Notification | PUN | 196148 | 688500 | 716315.4 | 745254.5422 | 775362.8257 | 806687.4838 |
| Case and outbreak verification and investigation | PUN | 0 | 561000 | 583664.4 | 607244.4418 | 631777.1172 | 657300.9127 |
| Data Management | PUN | 0 | 185785.86 | 192102.5772 | 138010.1004 | 209150.3346 | 149386.5711 |
| Laboratory | PUN | 0 | 38250 | 39795.3 | 41403.03012 | 43075.71254 | 44815.97132 |
| Supportive Activities | PUN | 0 | 0 | 0 | 0 | 0 | 0 |
| Surveillance and Monitoring | FAT | 0 | 111180 | 115671.672 | 120344.1708 | 125204.9279 | 130262.7654 |
| Detection and Notification | FAT | 0 | 45900 | 47754.36 | 49683.63614 | 51690.46537 | 53778.67184 |
| Case and outbreak verification and investigation | FAT | 0 | 36720 | 38203.488 | 39746.48443 | 41352.15581 | 43022.71666 |
| Data Management | FAT | 0 | 28560 | 29713.824 | 30914.05025 | 32162.30677 | 33461.3769 |
| Laboratory | FAT | 0 | 0 | 0 | 0 | 0 | 0 |
| Supportive Activities | FAT | 0 | 0 | 0 | 0 | 0 | 0 |
| Surveillance and Monitoring | CDA | 0 | 8772 | 29289.3408 | 30472.63017 | 31703.55124 | 32983.84475 |
| Detection and Notification | CDA | 0 | 3060 | 3183.624 | 3312.24241 | 3446.057003 | 3585.277706 |
| Case and outbreak verification and investigation | CDA | 0 | 3672 | 3820.3488 | 3974.690892 | 4135.268404 | 4302.333247 |
| Data Management | CDA | 0 | 2040 | 22285.368 | 23185.69687 | 24122.22583 | 25096.2338 |
| Laboratory | CDA | 0 | 0 | 0 | 0 | 0 | 0 |
| Supportive Activities | CDA | 0 | 0 | 0 | 0 | 0 | 0 |
| Surveillance and Monitoring | SIN | 22000 | 1122558.96 | 677249.4204 | 528147.3035 | 538926.736 | 549930.5032 |
| Detection and Notification | SIN | 8842 | 204728.28 | 208822.8456 | 212999.3025 | 217259.2886 | 221604.4743 |
| Case and outbreak verification and investigation | SIN | 0 | 153000 | 156060 | 159181.2 | 162364.824 | 165612.1205 |
| Data Management | SIN | 13158 | 105807.66 | 190669.9464 | 31836.24 | 32689.45123 | 33568.47274 |
| Laboratory | SIN | 0 | 0 | 0 | 0 | 0 | 0 |
| Supportive Activities | SIN | 0 | 659023.02 | 121696.6284 | 124130.561 | 126613.1722 | 129145.4356 |

Table A3: Model Fitting and Transformations

The coding relies on HydeNet implemented in the R programming library [(Team and Others 2013; Nutter )](https://paperpile.com/c/nP6BzD/wvFT+qkMP). We provide basic likelihoods of priors here for clarity in understanding the model.

### Bayesian model fitting and code

Variable names

MandS.popscaled = Monitoring and Surveillance spending, ln+1

SiaBud.popscaled = Supplemental immunization budget, ln+1

QualRank=Risk Rank

sumAFP=Acute Flaccid Paralysis Cases, ln+1

world_pop_u15=District Population, under 15, ln+1

Polio_Case_Count= Count of polio cases, ln+1

NeighbCase=Case in neighboring district

RiskScore=calculated probability of polio case

MopUpm=mop up campaign immunizations

NIDm=National campaign immunizations

SNIDm= Supplemental immunization campaigns

Type1.Immunity.t2= Type one immunity, next time period

raised_priority=decrease in risk ranking (table A1)

lowered_priority=decrease in risk ranking (table A1)

##

##

## Model results with uncertainty.

|  | **Monitoring and Surveillance Spending** | | |
| --- | --- | --- | --- |
| *Predictors* | *Estimates* | *CI* | *p* |
| (Intercept) | -6.49 | -8.50 – -4.47 | **<0.001** |
| QualRank | 0.27 | 0.08 – 0.47 | **0.007** |
| Year [2014] | 2.81 | 2.28 – 3.34 | **<0.001** |
| Year [2015] | 2.81 | 2.30 – 3.33 | **<0.001** |
| Year [2016] | 3.01 | 2.50 – 3.52 | **<0.001** |
| SiaBud.popscaled | 1.00 | 0.84 – 1.15 | **<0.001** |
| Observations | 272 | | |
| R^2^ / R^2^ adjusted | 0.613 / 0.606 | | |

|  | **AFP** | | |
| --- | --- | --- | --- |
| *Predictors* | *Estimates* | *CI* | *p* |
| (Intercept) | -294.74 | -355.10 – -234.37 | **<0.001** |
| MandS.popscaled | 1.69 | -1.17 – 4.56 | 0.246 |
| SiaBud.popscaled | 4.54 | -5.02 – 14.10 | 0.350 |
| Year [2014] | -4.12 | -19.69 – 11.44 | 0.602 |
| Year [2015] | -3.58 | -18.33 – 11.18 | 0.633 |
| Year [2016] | 17.29 | 3.04 – 31.54 | **0.018** |
| world_pop_u15 | 19.91 | 8.95 – 30.86 | **<0.001** |
| Province [fata] | 29.85 | 15.86 – 43.85 | **<0.001** |
| Province [islamabad] | 20.93 | -16.26 – 58.12 | 0.269 |
| Province [kp] | 24.30 | 10.76 – 37.84 | **<0.001** |
| Province [punjab] | 48.19 | 30.68 – 65.71 | **<0.001** |
| Province [sindh] | -3.60 | -18.71 – 11.51 | 0.640 |
| Observations | 272 | | |
| R^2^ / R^2^ adjusted | 0.668 / 0.654 | | |

|  | **Raised Priority** | | |
| --- | --- | --- | --- |
| *Predictors* | *Odds Ratios* | *CI* | *p* |
| (Intercept) | 0.04 | 0.00 – 0.39 | **0.007** |
| Polio_Case_Count | 0.88 | 0.71 – 0.99 | 0.137 |
| NeighbCase [1] | 1.23 | 0.60 – 2.65 | 0.584 |
| Type1.Immunity.t2 | 16.13 | 0.94 – 355.03 | 0.065 |
| Observations | 272 | | |
| R^2^ Tjur | 0.030 | | |

|  | **Lowered Priority** | | |
| --- | --- | --- | --- |
| *Predictors* | *Odds Ratios* | *CI* | *p* |
| (Intercept) | 0.23 | 0.02 – 2.00 | 0.197 |
| Polio_Case_Count | 0.88 | 0.71 – 0.99 | 0.118 |
| NeighbCase [1] | 2.78 | 1.12 – 8.43 | **0.043** |
| Type1.Immunity.t2 | 0.38 | 0.02 – 6.87 | 0.500 |
| Observations | 272 | | |
| R^2^ Tjur | 0.028 | | |

## JAGS Model Code

| model{ |
| --- |
| MandS.popscaled ~ dnorm( -6.48667 + 0.27221*QualRank + 2.81083 + 2.81385 + 3.00934 + 0.99701*SiaBud.popscaled, 0.666529554404763) |
| QualRank ~ dnorm( 0, 1) |
| pi.Year[1] <- 0.2; pi.Year[2] <- 0.2; pi.Year[3] <- 0.2; pi.Year[4] <- 0.2; pi.Year[5] <- 0.2 |
| Year ~ dcat(pi.Year) |
| SiaBud.popscaled ~ dnorm(13.57529 + 0.53148 + 0.19554 + 1.03875 + 0.07977 + -1.23149 + 0.65333 + 0.82791 + 1.80823 + -2.73406 + -2.72708 + -1.86686 + -1.12276 + -2.90379 + -2.52168 + 0.00098 + -0.29483 + 0.37866 + -2.76012 + -0.32807 + -0.3446 + -0.57853 + -1.70955 + -0.53798 + -0.09442 + 0.09276 + -0.29484 + 0.40668 + -0.40875 + -0.00439 + 0.33047 + -1.17904 + -0.35666 + 1.7127 + -0.38799 + -0.03463 + -0.7048 + -0.75205 + 1.28366 + -0.73763 + 0.18555 + -0.58991 + 1.31616 + 1.19279 + 0.05814 + 0.72064 + -0.93269 + 1.58111 + |
| -0.54206 + 0.94254 + 0.23891 + 1.25115 + 1.48374 + 0.7603 + 0.34561 + 0.04685 + -2.04091 + 1.42197 + -0.15381 + -0.12695 + 0.93697 + 1.1523 + -0.49968 + -0.79657 + 0.60977 + -0.50579 + -0.48542 + -1.17921 + -0.19254 + -0.36453, 11.23313) |
| sumAFP2 <- sumAFP^2 |
| sumAFP ~ dnorm( -294.73716 + 1.69073*MandS.popscaled + 4.54087*SiaBud.popscaled + -4.12492 + -3.57655 + 17.29136 + 19.90509*world_pop_u15 + 29.85175 + 20.93203 + 24.29939 + 48.19377 + -3.59899, 0.0342766976794814) |
| world_pop_u15 ~ dnorm(12.56216, 0.73022) |
| pi.Province[1] <- 0.191176470588235; pi.Province[2] <- 0.176470588235294; pi.Province[3] <- 0.0147058823529412; pi.Province[4] <- 0.220588235294118; pi.Province[5] <- 0.176470588235294; pi.Province[6] <- 0.220588235294118 |
| Province ~ dcat(pi.Province) |
| Polio_Case_Count ~ dpois(exp(-18.54757 + -0.31259 * Stool.Adequacy + 0.01749 * sumAFP + -6e-05 * sumAFP2 + 0.8507 * RiskScore + 0.44513 + 0.78558 + -0.28571 + -0.63384 + 18.25264 * NeighbCase)) |
| Stool.Adequacy ~ dnorm(0.85041, 66.57552) |
| RiskScore ~ dnorm(-0.86806, 0.70683) |
| NeighbCase ~ dbern( ilogit(1.68151 + 0.43285*RiskScore + 2.01856 + -2.08104 + 1.65672 + -0.85443 + 0.11357 + 0.2091 + 0.53755 + -0.13164 + -0.97538)) |
| pi.District[1] <- 0.0147058823529412; pi.District[2] <- 0.0147058823529412; pi.District[3] <- 0.0147058823529412; pi.District[4] <- 0.0147058823529412; pi.District[5] <- 0.0147058823529412; pi.District[6] <- 0.0147058823529412; pi.District[7] <- 0.0147058823529412; pi.District[8] <- 0.0147058823529412; pi.District[9] <- 0.0147058823529412; pi.District[10] <- 0.0147058823529412; pi.District[11] <- 0.0147058823529412; pi.District[12] <- 0.0147058823529412; pi.District[13] <- 0.0147058823529412; pi.District[14] <- 0.0147058823529412; pi.District[15] <- 0.0147058823529412; pi.District[16] <- 0.0147058823529412; pi.District[17] <- 0.0147058823529412; pi.District[18] <- 0.0147058823529412; pi.District[19] <- 0.0147058823529412; pi.District[20] <- 0.0147058823529412; pi.District[21] <- 0.0147058823529412; pi.District[22] <- 0.0147058823529412; pi.District[23] <- 0.0147058823529412; pi.District[24] <- 0.0147058823529412; pi.District[25] <- 0.0147058823529412; pi.District[26] <- 0.0147058823529412; pi.District[27] <- 0.0147058823529412; pi.District[28] <- 0.0147058823529412; pi.District[29] <- 0.0147058823529412; pi.District[30] <- 0.0147058823529412; pi.District[31] <- 0.0147058823529412; pi.District[32] <- 0.0147058823529412; pi.District[33] <- 0.0147058823529412; pi.District[34] <- 0.0147058823529412; pi.District[35] <- 0.0147058823529412; pi.District[36] <- 0.0147058823529412; pi.District[37] <- 0.0147058823529412; pi.District[38] <- 0.0147058823529412; pi.District[39] <- 0.0147058823529412; pi.District[40] <- 0.0147058823529412; pi.District[41] <- 0.0147058823529412; pi.District[42] <- 0.0147058823529412; pi.District[43] <- 0.0147058823529412; pi.District[44] <- 0.0147058823529412; pi.District[45] <- 0.0147058823529412; pi.District[46] <- 0.0147058823529412; pi.District[47] <- 0.0147058823529412; pi.District[48] <- 0.0147058823529412; pi.District[49] <- 0.0147058823529412; pi.District[50] <- 0.0147058823529412; pi.District[51] <- 0.0147058823529412; pi.District[52] <- 0.0147058823529412; pi.District[53] <- 0.0147058823529412; pi.District[54] <- 0.0147058823529412; pi.District[55] <- 0.0147058823529412; pi.District[56] <- 0.0147058823529412; pi.District[57] <- 0.0147058823529412; pi.District[58] <- 0.0147058823529412; pi.District[59] <- 0.0147058823529412; pi.District[60] <- 0.0147058823529412; pi.District[61] <- 0.0147058823529412; pi.District[62] <- 0.0147058823529412; pi.District[63] <- 0.0147058823529412; pi.District[64] <- 0.0147058823529412; pi.District[65] <- 0.0147058823529412; pi.District[66] <- 0.0147058823529412; pi.District[67] <- 0.0147058823529412; pi.District[68] <- 0.0147058823529412 |
| District ~ dcat(pi.District) |
| SNIDm ~ dnorm( -1535.77906 + 128.18212*SiaBud.popscaled + 38.76039 + 200.69901 + 27.88486 + 83.8786 + 41.81679, 0.00329380830427132) |
| MopUpm ~ dnorm( 2.8946 + 0.21244*Polio_Case_Count, 0.0971887411981457) |
| Type1.Immunity.t2 ~ dnorm( 0.68008 + -4e-05*MopUpm + 0.00012*SNIDm + -0.00024*NIDm, 8.66021047662242) |
| NIDm ~ dnorm(-4.32704, 0.03468) |
| raised_priority ~ dbern( ilogit(-3.11845 + -0.12839*Polio_Case_Count + 0.20589*NeighbCase + 2.78063*Type1.Immunity.t2)) |
| lowered_priority ~ dbern( ilogit(-1.45607 + -0.13053*Polio_Case_Count + 1.02218*NeighbCase + -0.97068*Type1.Immunity.t2)) |
| } |

## Directed Acyclic Graph


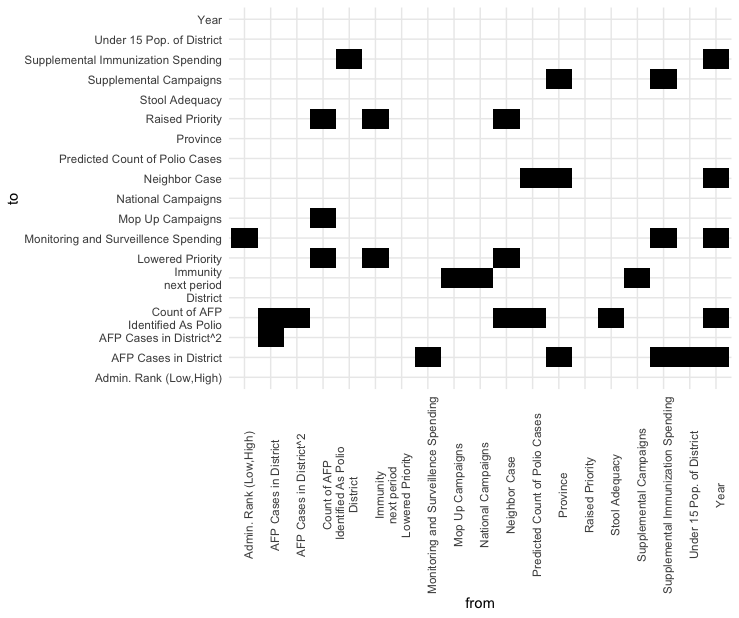


## Presented simulations

Figure 4 results based on compiling a model , fixing spending at $1 and $11.51 and simulating outcomes for AFP, polio cases, and risk ranking. Each point or observation represents outcome measures based on 10,000 results for each level of spending.

| dc4<-HydeSim(HydeNet::compileJagsModel(Net,data=list(MandS.popscaled=log(1)),n.chains=20),c("Polio_Case_Count","sumAFP","lowered_priority","raised_priority"),n.iter=10000)  dc5<-HydeSim(HydeNet::compileJagsModel(Net,data=list(MandS.popscaled=log(100001)),n.chains=20),c("Polio_Case_Count","sumAFP","lowered_priority","raised_priority"),n.iter=10000) |
| --- |

Figure 6 presents simulations fixing AFP rates at 1/100000 compared to 4/100000.

| lower_rate<-HydeSim(HydeNet::compileJagsModel(Net,data=list(world_pop_u15=mean(exp(ls1$world_pop_u15)),sumAFP=ceiling(mean(exp(ls1$world_pop_u15))/100000*1)),n.chains=20),c("RiskScore","Polio_Case_Count","sumAFP","Env.Samples.Taken","QualRank","SiaBud.popscaled","QualRankChange","lowered_priority","raised_priority","MandS.popscaled",'world_pop_u15'),n.iter=10000)  higher_rate<-HydeSim(HydeNet::compileJagsModel(Net,data=list(world_pop_u15=mean(exp(ls1$world_pop_u15)),sumAFP=ceiling(mean(exp(ls1$world_pop_u15))/100000*4)),n.chains=20),c("RiskScore","Polio_Case_Count","sumAFP","Env.Samples.Taken","QualRank","SiaBud.popscaled","QualRankChange","lowered_priority","raised_priority",'MandS.popscaled','world_pop_u15'),n.iter=10000)  rbind(lower_rate,higher_rate) %>%ddply(.,.(chain_index,Polio_Case_Count,sumAFP),summarize,"height"=length(raised_priority)) %>% ggplot()+geom_boxplot(aes(x=ifelse(Polio_Case_Count>15,15,Polio_Case_Count),y=log(height),colour=as.factor(sumAFP),group=interaction(ifelse(Polio_Case_Count>15,15,Polio_Case_Count),sumAFP)),position="identity",fill=NA)+theme_pander()+xlab("Polio Case Count")+ylab("Ln frequency in each Chain (10000)")+scale_colour_wsj(name="AFP Rate",labels=c("1/100000","4/100000")) |
| --- |

Figure 7 presents simulating polio case counts and lowering v. increasing risk rank of district.

| tencases1<-HydeSim(HydeNet::compileJagsModel(Net,n.chains=20),c("RiskScore","Polio_Case_Count","sumAFP","Env.Samples.Taken","QualRank","SiaBud.popscaled","QualRankChange","lowered_priority","raised_priority","MandS.popscaled",'world_pop_u15'),n.iter=100000)  tout1<-ddply(tencases1,.(ifelse(Polio_Case_Count>15,15,Polio_Case_Count),chain_index),summarize,"lowered"=sum(lowered_priority)/length(lowered_priority),"raised"=sum(raised_priority)/length(raised_priority))  colnames(tout1)[1]<-"Polio_case"  ggplot(tout1)+geom_boxplot(aes(x=as.factor(Polio_case),y=lowered,colour="lowered"))+geom_boxplot(aes(x=as.factor(Polio_case),y=raised,colour="raised"))+theme_pander()+xlab("Polio Cases")+ylab("proportion of districts with case count lowered/raised")+scale_colour_colorblind(name="priority rank") |
| --- |
